# Clinical and Epidemiologic Characteristics of Severe Childhood Ocular Injuries in Southern Iran

**DOI:** 10.4103/0974-9233.80702

**Published:** 2011

**Authors:** Hamid Hosseini, Masoumeh Masoumpour, Fatemeh Keshavarz-Fazl, M. Reza Razeghinejad, Ramin Salouti, Mohammad Hosein Nowroozzadeh

**Affiliations:** Poostchi Ophthalmology Research Center, Shiraz School of Medicine, Shiraz University of Medical Sciences, Shiraz, Iran

**Keywords:** Child Health, Epidemiology, Eye, Injury, Trauma

## Abstract

**Purpose:**

To evaluate the clinical and epidemiological characteristics of children with ocular trauma.

**Materials and Methods:**

We retrospectively reviewed the medical records of 278 children (aged 15 years or less) hospitalized with ocular injuries and treated as inpatients at a tertiary referral center in Shiraz, Iran, from 2005 to 2008. Nominal variables were evaluated with a Chi-square test. A *P*-value less than 0.05 indicated statistical significance.

**Results:**

The cohort was comprised of 205 (74%) males, outnumbering females by a ratio of 2.81/1. The mean age was 7.6 ± 3.96 years. Rural residents comprised 125 (45%) of the cohort. Sharp objects caused ocular injury in 211 (76%) cases, and 207 (74%) cases had open-globe injuries. The lens was injured in 62 (30%) cases at initial examination and 89 (43%) patients according to ultrasound examination (*P* = 0.006). Twenty-eight cases (10%) developed post-traumatic endophthalmitis. Endophthalmitis was associated with needle injury [odd ratio (OR) = 19.25] and presence of intraocular foreign body (OR = 3.48). Visual acuity of patients with closed-globe injuries was 20/200 or better on both initial and final examinations. Visual acuity of patients with open-globe injuries were in the range of light perception to 20/200.

**Conclusions:**

Trauma is an important cause of childhood ocular morbidity in southern Iran. Playing with sharp objects is an important cause of ocular trauma in children, and most injuries can be prevented by careful supervision.

## INTRODUCTION

Twenty percent to fifty percent of all ocular injuries^1^ occur in children. Ocular injury is a leading cause of monocular visual disability and non-congenital unilateral blindness in children.^2^ Additionally, the management of ocular injuries in children is complicated by the lack of cooperation during assessment and poor compliance with therapy. Although complications associated with severe injuries are well documented, the epidemiology of the traumatic events is not well defined. Additionally, the etiology of pediatric ocular injuries is likely to differ from adult ocular injury, and it requires further investigation.^1^

The purpose of this study is to describe the epidemiologic and clinical characteristics of ocular trauma in a large group of children (15 years of age or younger) who were admitted to a tertiary referral hospital in Iran during a 3-year period. A secondary aim was to describe the clinical course and outcomes, particularly with respect to infection and visual acuity.

## MATERIALS AND METHODS

We retrospectively reviewed the medical records of all children 15 years old and younger who were admitted to the hospital at our tertiary referral center (Khalili Hospital, Shiraz, Iran) from March 2005 to February 2008. This public hospital is the only tertiary referral center for pediatric eye trauma in Shiraz, and it serves most areas of southern Iran as well. This study was performed in accordance with the 1964 Declaration of Helsinki and the subsequent amendments. The study protocol was approved by the Ethics Committee of the Shiraz University of Medical Sciences, Iran.

Cases were defined according to the International Classifications of Diseases, Ninth Revision Codes, published by the World Health Organization. Data were retrieved from admission charts, surgical records and outpatient clinic files. Data included demographic information, initial and final visual acuity (VA), object causing the injury, type and location of laceration, status of the crystalline lens, other anterior and posterior segment findings at initial examination, primary and additional surgical procedures and complications. We also recorded the presence or absence and location of intraocular foreign bodies and other findings noted by examination, ocular or other imaging techniques. The month of injury was tabulated to identify possible seasonal variations.

VA was recorded as Snellen acuity when possible, according to the patient’s age and cooperation. VA was classified as category 1 (≥20/200), category 2 (<20/200 to light perception) or category 3 (no light perception).

According to the ocular trauma classification system,^3^ mechanical injuries of the globe were divided into “open globe” or “closed globe” injuries. An open-globe injury was defined as a full-thickness wound of the eyeball. A closed-globe injury was defined as either a contusion (defined as no corneal or scleral wound), a lamellar laceration (a partial thickness wound) or a superficial foreign body.

Statistical analysis was performed using SPSS software version 16.0 (SPSS Inc., Chicago, IL, USA). Nominal variables were evaluated with the Chi-square test and odd ratios (OR) were calculated. A *P*-value less than 0.05 was statistically significant.

## RESULTS

**Figure 1:**
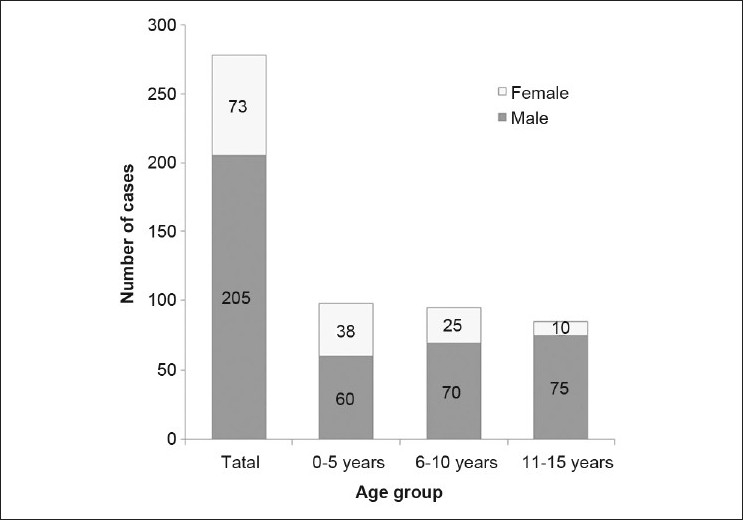
Age and sex of children hospitalized with eye injuries

Demographic characteristics of the cohort are presented in Figure 1. The mean age was 7.60 ± 3.96 years (range 9 months to 15 years, median 7 years). One-hundred twenty-five patients (45%) were from the surrounding rural communities. The number of hospitalizations for pediatric eye injury did not differ significantly between the age groups [(*P* = 0.78; Figure 1). There were a greater number of male patients, with a male-to-female ratio of 2.81:1. This male predominance significantly increased with advancing age (*P* < 0.001; Figure 1).

**Figure 2:**
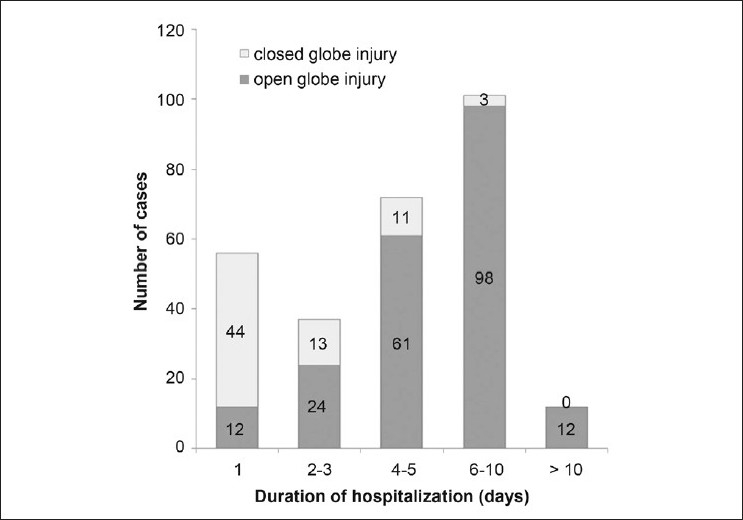
Duration of hospitalization for patients with open-globe or closed-globe eye injuries

The mean duration of hospitalization was 5 ± 3.35 days (range, 0-21 days). Figure 2 presents the duration of hospitalization for open- and closed-globe injuries. Most of the patients in the closed-globe injury group (65%) were admitted for only 1 day. About half of the patients with open eye injury were hospitalized between 6 and 10 days. Twelve (6%) patients in this group were admitted for more than 10 days, whereas no patients with closed eye injuries were admitted for this length of time.

**Figure 3:**
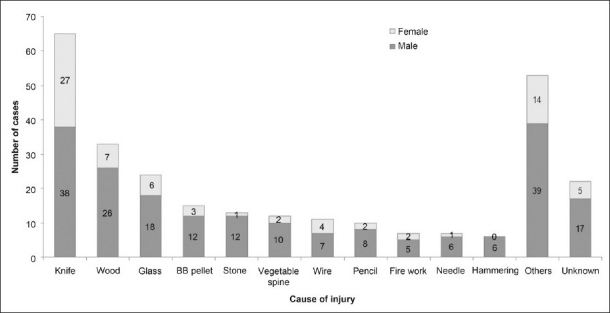
Objects causing eye injuries in children hospitalized for eye trauma

Most cases of ocular injury were caused by sharp objects (210, 76%), followed by blunt objects (67, 24%) (one case was ambiguous as we could not determine whether the insulting agent was sharp or blunt) [Figure 3]. While there was no significant difference in the number of ocular trauma cases by season (*P* = 0.19), there were more cases than expected during the Iranian celebration of Charshanbe-soorei (“Wednesday Eve Festival”), the last Tuesday night of the Persian calendar. The occasion is associated with injuries related to sharp objects and fireworks.^4^ 

Open-globe injuries were present in 207 (74%) patients. Among the open-globe injuries, the cornea was the only site of injury in most of the cases (134, 65%), followed by corneoscleral (43, 21%) and scleral injuries (26, 13%). (Location of the laceration was not specified in four cases.) Uveal prolapse occurred in 150 (72%) of the open-globe injuries. The lens was involved in 62 (30%) patients at initial examination and 89 (43%) subjects according to sonographic reports (*P* = 0.006). Posterior segment involvement was reported in 94 (45.4%) of the patients with open globe and in six (8.5%) patients with closed-globe injuries (*P* < 0.001).

The most common types of closed-globe injuries were lamellar laceration (68, 96%), followed by lid and periorbital lacerations (16, 22%), contusion (3, 4%) and superficial foreign bodies (3, 4.2%). Some cases suffered from mixed injuries. Significant lens involvement was found in only one case.

Retained foreign body was found in 12% (36) of the patients. The most common form was intraocular (19, 7%), followed by corneal (9, 3%), intraorbital (5, 2%) and conjunctival (3, 1%) foreign bodies.

Post-traumatic endophthalmitis developed in 28 (14%) cases with open-globe injury, with rural patients accounting for about half of these cases. An intraocular foreign body (IOFB) was detected in six cases of infection. The presence of IOFB was associated with a significant increase in the rate of post-traumatic endophthalmitis (OR = 3.48, *P* = 0.016). Trauma caused by injection needles was the leading cause of endophthalmitis (five of seven cases), and all cases had poor prognosis. This type of injury substantially increased the risk of post-traumatic endophthalmitis compared with the other types of open-globe injury (OR = 19.25, *P* < 0.001). Other common objects causing injuries that led to endophthalmitis were knives (four cases), wires (four cases) and vegetable spines (three cases). Endophthalmitis was managed by an intravitreal injection of antibiotics (vancomycin 1 mg/0.1 ml plus ceftazidime 2.5 mg/0.1 ml) in nine cases and core vitrectomy in 18 cases. One case needed revision of deep vitrectomy twice (due to total retinal detachment and emulsification of silicone oil) and another case required enucleation.

**Figure 4:**
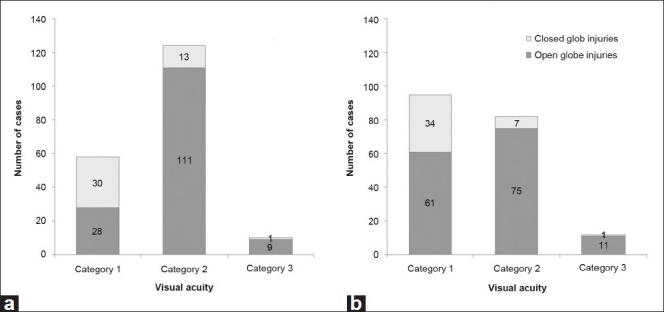
Initial (a) and final (b) visual acuities in children with open- and closed-globe injuries. (Category 1, ≥20/200; Category 2, <20/200 to light perception; Category 3, no light perception)

Of the total 278 patients, 86 children were too young to obtain Snellen VA and were recorded as having or not having CSM (Central, Steady and Maintain). Of the remaining 192 patients, all had initial VAs recorded, and 189 cases had final VAs recorded. Figure 4 demonstrates that in the closed-globe injury group, the VA is stable between initial and final measurements. Most of the patients in this group had both initial and final VA of 20/200 or better. On the other hand, most of the patients with open-globe injuries had an initial VA of less than 20/200 to light perception. A large percent of the patients with open-globe injuries improved to category one (VA ≥20/200) by the time they were discharged. Most (10, 83%) of the patients who became blind had no light perception at initial examination. Nearly all the patients who became blind (11, 92%) had an open-globe injury.

Overall, 257 (92%) cases underwent surgery. Of those with closed-globe injuries, 63 cases (89%) required surgical treatment. Most procedures in this group were diagnostic peritomy (26, 41%), followed by repair of conjunctival, partial thickness corneal or scleral laceration (25, 40%), repair of lid laceration (16, 25%) and removal of superficial foreign bodies (3, 5%). Six cases underwent a second operation for suture revision (three cases), enucleation (one case), lensectomy and intraocular lens (IOL) insertion (one case) and anterior chamber irrigation (one case). Of those with open-globe injuries, 194 (94%) children received surgical intervention and 13 (6%) were observed to follow self-sealing injuries. More than one surgical procedure was performed in 126 (65%) of the patients. Some underwent multiple procedures: two procedures (73, 38%), three procedures (34, 18%) and four procedures (10, 5%).

Lensectomy was performed in 28% (73) of the surgical cases and intraocular lens was implanted in 33 (45%) patients. Deep vitrectomy was performed in 52 (20%) cases that underwent surgical procedures. The most prevalent indication for vitrectomy was retinal detachment (25, 48%), followed by vitreous hemorrhage (21, 40%), endophthalmitis (6, 31%), IOFB (9, 17%) and macular hole (1, 2%). Enucleation was performed in 11 cases (4%) that required surgery.

## DISCUSSION

Ocular trauma can be a devastating injury in children and may cause lifelong disability. Visual loss due to these injuries can be mitigated or even avoided completely through prevention and through parental supervision and education of children, parents and care givers on the use of protective eyewear during high-risk activities.^5^

Several limitations of this study should be considered when the outcomes are compared with previous publications.^1^,^6^–^15^ This study is limited by its retrospective nature. Moreover, the data include only information related to injuries that required hospitalization. Our findings are not representative of eye injuries that did not receive medical attention, injuries treated in other health care settings or injuries that did not require hospitalization. Thus, our results do not show the overall burden of pediatric eye injuries in the population. Further studies can help determine the overall burden by integrating data from hospitals, emergency rooms and outpatient clinics.

In this study, the distribution of hospitalization for pediatric (≤15 years of age) eye injury showed no significant trends with age. This outcome is similar to the results of the study by Nelson and associates.^16^ However, other studies have found increasing ocular injuries with age.^17^–^19^ The differences in observations could result from demographic and cultural differences.

We found that the number of male patients hospitalized for eye trauma was twice that of females. This finding is consistent with previous research, indicating that males have a significantly higher frequency of pediatric eye injuries.^7^,^8^,^10^,^15^,^20^ Furthermore, the difference between the sexes increased considerably among the older age groups [Figure 1]. This disparity between sexes seems to be related to a more aggressive behavior in males as well as a greater participation in high-risk activities compared with females.^13^

In this study, the most common type of pediatric eye injury resulting in hospitalization was open-globe injuries. This finding is similar to previous studies conducted in the United States,^7^ Jordan^21^ and Turkey.^15^,^22^ However, other investigators found that greater hospitalization occurred in closed-globe injuries cases compared with cases with open-globe injuries.^20^,^23^,^24^ It should be noted that these results do not imply that open-globe injuries are more frequent than closed-globe injuries. Most of the children with closed-globe injuries are treated on an outpatient basis. The small fraction of this group that was hospitalized includes the most severe cases in this category. On the other hand, nearly all subjects with open-globe injury upon diagnosis are admitted to the hospital for close observation and systemic antibiotics as a minimal precaution.

An important finding in our study was the number of injuries caused by the use of sharp objects as toys. We believe that this may be a common problem in developing countries. The most common objects leading to open-globe injuries were knives, followed by wood and glass [Figure 3]. Perforation with knives usually occurred accidentally, during unsupervised play. Encouraging increased parental supervision and decreased access to sharp objects could decrease the childhood ocular injuries. However, preventive strategies revolving around public education may be of limited value.^25^ Therefore, other strategies may be required. Given that knives constituted a large proportion of objects causing eye injuries, replacing sharp knives with round blunt-tipped knives might also help prevent these injuries.

Although wearing automobile seatbelts is required by law, they are not widelyx used and children often sit in the front or back seat unrestrained. As a result, when they are involved in traffic accidents, ocular injuries due to flying glass particles are more likely. Consequently, new legislation that mandates wearing seatbelts in the back seat may reduce eye injury during motor vehicle accidents. Other legislation that mandates children participating in high-risk sports or jobs to use protective eyewear may also be of value.

We found that needle injury and presence of intraorbital foreign bodies were significantly associated with post-traumatic endophthalmitis. Soylu *et al*.^8^ reported that all children with injection needle injuries to the eye had endophthalmitis on admission in southern Turkey. Because many patients with ocular needle injury may not seek ophthalmic care, or may be treated at primary health care centers, this high rate of endophthalmitis may be overestimated to some extent by referral bias. Another possible explanation may be greater contamination or deeper penetration of needles into the eye.

Our study revealed that ultrasonography was more sensitive than biomicroscopy in detecting lens involvement in pediatric eye trauma. This finding may be due to difficulties in examining small children. Therefore, it would be prudent to use ultrasonography for ruling out lens involvement whenever clinical examination is inconclusive or impossible.

In summary, the results of our study indicate that a substantial number of children are hospitalized for ocular trauma in this part of Iran. Although we need to improve our quality of primary surgery and treatment of open-globe trauma, a special focus on the prevention of injury is warranted.
